# Supplementary material for: Bacteriological quality and antimicrobial susceptibility profiles of isolates of ready-to-eat raw minced meat from hotels and restaurants in Arba Minch, Ethiopia
Source: PLoS One. 2022 Sep 1;17(9):e0273790. doi: 10.1371/journal.pone.0273790 (PMC9436051; doi:10.1371/journal.pone.0273790)
Supplement: Attachment — Submitted filename: Reviewer Comments PONE-D-22-11268.docx [file pone.0273790.s001.docx]

The manuscript PONE-D-22-11268 entitled “*Bacteriological quality and antimicrobial susceptibility patterns of isolates of ready-to eat raw minced meat from hotels and restaurants in Arba Minch, Ethiopia*” provides insights into the bacteriological quality of ready-to-eat raw minced meat in Arba Minch in Eithopia. It is worth mentioning that this type of study is important in places where people consume raw meat also as is the case in cuurent scenario. The study is all the more important as many isolates were multi drug resistance which is a grim situation. However, the manuscript has some major lacunae and shortcomings, which should be considered before its approval.

**Abstract:** Make it more concise, staring from a cross sectional study…Include lines on MDR and MRSA also.

**INTRODUCTION**

The Introduction includes many grammatical errors. The following sentences need to re-written as below.

Line 57: Ethiopia ranks second after Nigeria in this regard and FBDs pose a serious threat to the health of the people in the country.

Line 67: The most common source of the bacterial causes of diarrheal diseases via meat chains is food of animal origin, environment, meat handlers/processors and processing equipment.

Line 69: Even though the source of the bacteria varies, raw meat is one of food of animal origin which has been confirmed as one of the common vehicles of foodborne diseases.

Line 72: Moreover, The most common food safety problems …….

Line 78: Transmission of these resistant bacteria to humans via meat is most evident ………

Line 84: The problem is even more alarming in developing countries, where there is an

enormous burden of infectious diseases, accompanied by lack of surveillance networks, paucity of testing laboratories, and inadequate diagnostics.

Line 87: Although healthy meat from beef cattle, sheep and goat …

Line 91: The general masses comprising all age groups of the study area, Arba Minch town….

Line 93: Nevertheless, many hotels and restaurants are also serving ready-to-eat raw meats, without assessing its bacteriological quality, which is further aggravated by lack food safety and inspection department in the town.

Line 95: Literature survey indicates that studies in this context are sorely lacking.

**MATERIALS AND METHODS**

The study was carried out for a period of six months. However, the samples were collected for only 2.5 months and that too has been wrongly mentioned as 01 July to 31 Oct (Line 116), which comes out to be four months. Kindly rectify it and remain consistent throughout the text and tables for the period of study.

Line 105: The number of animals slaughtered per day varied from time to time, for instance, during fasting, festival and non-fasting periods.

Line 106: The average number of cattle slaughtered per month approximates to 600 excluding sheep and goats.

Line 109: Ethical approval for this study was obtained from the Institutional Review Board of College of Medicine and Health Science, Arba Minch, Eithopia (Ref. No IRB/174/12/17/03/2020).

Line 117: A total of 260 samples were randomly collected comprising 170 beef, 50 sheep and 40 goat meat samples.

Line 118: One beef sample and two samples from goat meat……………..

Line 122: Rewrite

Line 125: Inclusion criterion was RTE raw minced meat (regional name of RTE: Kurt/Kitfo) that is processed for direct consumption, whereas the exclusion criterion was minced meat that is processed for cooking.

Line 130: Collected samples were drawn (from where…………..??????) by sterile tweezers and placed in a sterile plastic bag and immediately transported in ice box to the Medical Microbiology and Parasitology Laboratory, Department of Medical Laboratory Science for bacteriological analysis, within 2 h of collection.

Line 141: After incubation, plates showing between 30 and 300 colonies were counted and the results were calculated according to ISO 7218:2007 using the following formula [15] This ISO standard has been revised and the annex D pertains to *Salmonella*. The reference and the formula needs to be cross checked.

Line 153: The pdf file of reference 16 to be furnished please. Moreover, it’s a very old reference, maybe substituted with latest available.

Line 155: About 0.1ml from each dilution was spread on to the such as MaConkey agar and mannitol salt agar (Himedia, India) for the total coliform count and *S. aureus* count respectively and incubated at 37°C for 24 hours.

Line 159 and 164: use *were* instead of *was*

Line 171: *S. aureus* in italics

Line 183: Included

Line 196-197: *Salmonella* Typhimurium

**RESULTS**

Overall the data is poorly presented in the results.

Line 215: On the contrary, ~~respectively,~~ 13.2% (n=34), 17.5% (n=45) and 17.5% (n=45) of meat samples had unacceptable ranges of bacteriological load with respect to TVC, TCC and TSC, respectively. The word respectively has been wrongly written in the middle of the sentences at many places throughout the text. It should be mentioned at the end of the sentence (where ever required) after putting a comma. Should be changed and corrected throughout.

Line 218: The most alarming factor is that, 10.5% (n=4), 8% (n=4) and 1.8% (n=3) of beef, sheep and goat meat 219 samples respectively were contaminated with *S. aureus* and their TSC loads remained in a 220 potentially harmful range (Table 1)…. These figures donot match with the figures quoted against different species in Table No. 1

Line 221: *were* inplace of *are* and *was* inplace of *is*… correct the entire text for the grammatical errors..It has to be presented in past tense rather than present.

Line 223: use permissible instead of permitted

Line 245: six

Line 247: correct spellings of susceptibility

Line 258: In case of *E. coli* isolates, a higher degree……………

Line 269: 51%

Try replacing sheep and goat meats by mutton and chevon, respectively in text.

Table No.2 How can the TCC and TSC values for beef be lesser than sheep and goat meats, when the TVC is on the higher side?

Confirmation of isolates by biochemical characterization and molecular tests is very important, including identification of pathogenic isolates of the bacteria.

**DISCUSSION**

The discussion is not adequately written. The first page is a repetition of the results only. Simply comparisons have been made with other references in Eithopia. Only one global reference is there, which is Nepal. Comparison of the finding in meat may be done with that of human and animal prevalence too. Risk factors maybe identified to justify the statements made.

The discussion on AMR and MRSA should be more focused.
